# Supplementary material for: Adjustment for index event bias in genome-wide association studies of subsequent events
Source: Nat Commun. 2019 Apr 5;10:1561. doi: 10.1038/s41467-019-09381-w (PMC6450903; doi:10.1038/s41467-019-09381-w)
Supplement: Supplementary file 3 — Description of Additional Supplementary Files [file 41467_2019_9381_MOESM3_ESM.pdf]

Description of Additional Supplementary Files

Supplementary Data 1 :

Association statistics for 170 CD susceptibility SNPs in GWAS of prognosis
