# Supplementary material for: Adjustment for index event bias in genome-wide association studies of subsequent events
Source: Nat Commun. 2019 Apr 5;10:1561. doi: 10.1038/s41467-019-09381-w (PMC6450903; doi:10.1038/s41467-019-09381-w)
Supplement: Supplementary file 2 — peer review file [file 41467_2019_9381_MOESM2_ESM.pdf]

Reviewer #1 (Remarks to the Author):

Overview: This paper presents an approach to adjust for index event bias in genome-wide association studies (GWAS) of recurrent events. Unlike dealing with collider bias where adjustment for a variable in the pathway to the outcome of interest biases estimates of the genetic association because the variable is also associated with the genetic variant, this paper addresses the situation where investigators are examining genetic associations for recurrent events after conducting a GWAS of the primary event. For example, searching for genetic variants associated with recurrent heart attacks after conducting a GWAS on the primary heart attack. There are two ways in which the index event bias can occur: one can be through analyses of case-control studies with adjustment for the primary event; a second can be through analyses of cases only, considering only those who experienced the first event. The authors propose a new approach that uses the regression of the genetic effects on prognosis (subsequent events) on the genetic effects of incidence (primary event), estimating a slope that will be used for bias correction. In this regard it is similar to MR-Egger. This approach is derived from linear models for the incidence and recurrent (prognostic) events. However, the regression slope is biased and hence, the authors propose to correct for the bias through either a simulation approach or a Hedges-Olkin adjustment. With the bias-corrected estimate and its standard error, it is then possible to conduct hypothesis testing and obtain accurate p values, using the normal distribution. While the method seems generally appropriate, there are several issues that should be addressed.

Comments:

1. The main issue with the paper is that the simulations have been conducted under an unrealistic scenario in which traits are simulated as quantitative traits with 50% heritability and a non-genetic confounder explaining 40% of the variation in both incidence and prognosis. This scenario is highly unlikely. In all my years of conducting regression analyses, I have never seen such strong contributors to the variation in a trait. I guess it means that 90% of the trait is explained. While the authors admit that this is an unlikely scenario, they used it to be sure that the simulation "satisfies the assumptions of our procedure while creating a high degree of index event bias." Even with 65% of the variation in prognosis unexplained for each SNP (or explained by unmodeled confounders), the model is still unrealistic. Unfortunately, given this unrealistic scenario, the reader is unable to judge how important this issue is in the context of real world data. It would be useful to see some simulations for less extreme scenarios.

2. The models considered assume linearity between the outcomes and their predictors. However, the outcomes are inherently dichotomous (or survival outcomes). While the authors legitimately point out that the logistic model is nearly linear in portions of the predictor space, it would be more appropriate to address the underlying logistic model.

3. It seems that a significance level of 5% is used in most evaluations. This alpha level seems unusually high for GWAS studies.

4. Tables: the authors need to state what parameter/test the type I error and power are for. Is this the type I error and power for a single SNP test?

5. The authors mention the important assumption of the independence between direct genetic effects on prognosis and those on incidence. Is there any way around this assumption? It seems likely that some genetic variants are associated with both incidence and prognosis. Does your approach mean that only variants that are different from (and indeed independent of, including those in LD) those identified for incidence are eligible for association with prognosis? Would conditioning on these variants associated with incidence in the prognosis analysis deal with this issue, allowing for newly discovered variants to those independent of the known ones for incidence? Further it would provide for evaluation of the known ones in regard to incidence.

Reviewer #2 (Remarks to the Author):

This is an interesting manuscript that discusses bias in genetic association test of a secondary phenotypes posterior to a primary index event (e.g. survival, severity). The authors propose solving the estimation of direct genetic effect on the secondary phenotype through an adjustment procedure. The problem is fairly complex but relatively well stated, and it seems that the proposed approach at least helps reducing the bias. However, the importance of the problem in real data and the performances of the method remain unclear (see major comments below). Moreover, the real data applications provide suggestive support at most, but highlight a limited contribution of the proposed approach in solving the problem. My major comments are:

1) It feels like the authors choose a modelling that fit their theoretical solution. It makes the interpretation of performances difficult. In particular, equation (2) assumes Y is a function of X. However, the final scenario of interest is slightly different -- Y is a phenotype that is measurable only among cases, i.e. there’s no data for severity or survival among controls. It might be reasonable to build and demonstrate the relevance of the proposed solution using equation (2), but if the ultimate goal of the authors is to propose a solution applicable in real data, some changes are needed:

1a) the results from table 1 and 2 should be moved to the supplement. They are of limited interest as they relate to the approximation of equation (2). Results from table 3) and 4) are not fully satisfying either as they also use equation (2). Instead the primary table(s) should present results from simulations where X is binary and generated using an LTM/logistic model, and Y is generated in cases only using a model that depends on G and U but not on X.

1b) on top of effects of genetic correlation, it would be useful to have results showing the impact of disease prevalence, the distribution of the secondary outcome and minor allele frequency on the bias.

2) As showed in table 1 and 3, and as stated by the authors, the proposed method is imperfect, but likely reduces the bias as compared to a naïve approach that does not correct for anything. However, again, I feel the analysis is incomplete. In particular, as proposed by others, I ran a few simple simulations under the scenario described in comments 1a) and compared results of Y~G in cases only against Y~G+X in the complete sample (i.e. adjusting for the index event). Here I assumed Y is severity and positive only, setting Y to 0 for all controls. Overall, the latter solution perform clearly better than the case only analysis (i.e. most of the bias is removed). This solution is also imperfect as it can also induce a collider bias, but the question is, how much imperfect is it as compared to the proposed solution. Overall, my point is that the manuscript should include more method comparison, including the above strategy and any other potentially relevant approach.

3) The main assumption of the method is the absence of correlation between SNP effect on incidence and on prognosis. At the same time –based on the authors work– bias exists only if there is an overlap of risk factors between incidence and prognosis (the U variables, explaining up to 40% of the outcome's variances in some simulations). If I understood correctly, we end up in the weird situation where the method is of interest when there is correlation in risk factors (and therefore some bias), but only when this correlation is not due to genetic factors. If so, I expect the actual real case scenarios where the method is valid and useful to be quite narrow.

4) The IPF result is not very convincing and I do not see it as a proof of concept – at most it suggests that the approach may be of interest in situations where the effect of a SNP on index event is strong. The reported variability of the parameters depending on the approach used and the imputation quality threshold, and the huge confidence interval are particularly concerning. Statement such as "[we] have shown an example in IPF where a strong effect on susceptibility leads to a substantial index event" do not reflect the actual reported result. The only fair conclusion from this analysis – and a guideline for future study- is, in my opinion, that the method is not applicable for small sample size.

Other comments:

5) In the modelling section, the authors state that the approximation of a logistic/LTM model is valid as long as the SNP effects are small ("the small effect typical to polygenic traits.", page 18). However, in their first simulation example, they consider strong effect ("[…] ensures that the individual SNP effects are strong given the heritability.", page 7). Are these effects strong enough to impact the validity of the approximation? A few words on this would is welcome.

6) Page 6 – if I followed correctly, I think it should say "its effect on prognosis CONDITIONNAL ON X, beta_hat_prime[GY]…"

7) Page12 – Looks like a typo in the sentence “Some authors have argued that independently pleiotropic effects are likely to be the norm in complex disease”. Should “independent” be removed?

Reviewer #3 (Remarks to the Author):

Index event bias—which can occur when studying a trait that occurs after a primary event, notably when studying prognosis after disease diagnosis—is an important concern that often goes unacknowledged or is downplayed (“our study could be affected by index event bias, but the effect is probably small, and anyway there is nothing we can do about it”). This paper does a nice job explaining and highlighting this bias; placing it in the context of recent work on related issues (eg collider bias); and providing a clever procedure to actually adjust for index event bias. Through simulations and real data applications the authors show that their method can eliminate or reduce index event bias, and they give a feel for situations where this bias may be more or less of a concern.

One potential limitation is that the proposed methods are based on linear models for continuous phenotypes. This certainly makes the maths easier, and the authors show that the procedure performs reasonably well when applied to binary traits (notably disease incidence and prognosis). Nor is this the first time statistical geneticists apply least squared regression to non-continuous outcomes, decades of biostatistical tradition be damned. So this is fine—other subsequent papers can sort out whether other link functions help here.

Another issue, which the authors do not discuss but should, is censoring. Survival after diagnosis is usually a censored trait, which opens up additional pathways to index event bias. I suspect that there are plenty of opportunities for both incidence and censoring to be affected by a common confounder—eg when studying lung cancer survival. SNPs associated with smoking will be associated both with lung cancer incidence and censoring (eg death due to cardiac disease). It may be that the methods and simulations developed for a direct incidence/prognosis confounder work fine when incidence and censoring are confounded, but some explicit discussion of this would be nice.

A few minor points follow, that the authors may wish to address.

—investigators often construct a genetic risk score for incidence and then ask whether that is associated with prognosis. See eg PMID:24411283, Qi Guo. What are the implications of index event bias for these kind of analyses?

—if one is interested in predicting prognosis, should one adjust for index event bias? As predictors, the “biased” estimates (in the sense of not capturing causal effects) may be just fine. This might merit a comment.

Adjustment for index event bias in genome-wide association studies of subsequent events

Response to reviewers

Reviewer #1 (Remarks to the Author):

Overview: This paper presents an approach to adjust for index event bias in genome-wide association studies (GWAS) of recurrent events. Unlike dealing with collider bias where adjustment for a variable in the pathway to the outcome of interest biases estimates of the genetic association because the variable is also associated with the genetic variant, this paper addresses the situation where investigators are examining genetic associations for recurrent events after conducting a GWAS of the primary event. For example, searching for genetic variants associated with recurrent heart attacks after conducting a GWAS on the primary heart attack.

There are two ways in which the index event bias can occur: one can be through analyses of case-control studies with adjustment for the primary event; a second can be through analyses of cases only, considering only those who experienced the first event. The authors propose a new approach that uses the regression of the genetic effects on prognosis (subsequent events) on the genetic effects of incidence (primary event), estimating a slope that will be used for bias correction. In this regard it is similar to MR-Egger. This approach is derived from linear models for the incidence and recurrent (prognostic) events. However, the regression slope is biased and hence, the authors propose to correct for the bias through either a simulation approach or a Hedges-Olkin adjustment. With the bias-corrected estimate and its standard error, it is then possible to conduct hypothesis testing and obtain accurate p values, using the normal distribution.

While the method seems generally appropriate, there are several issues that should be addressed.

Comments:
1. The main issue with the paper is that the simulations have been conducted under an unrealistic scenario in which traits are simulated as quantitative traits with 50% heritability and a non-genetic confounder explaining 40% of the variation in both incidence and prognosis. This scenario is highly unlikely. In all my years of conducting regression analyses, I have never seen such strong contributors to the variation in a trait. I guess it means that 90% of the trait is explained.

It is uncontroversial that a trait can have 50% heritability dispersed among 10,000 SNPs – similar models have been previously inferred from data and used for simulations [1-3]. (Note that this is the variation explained by true genetic effects, not by effects estimated by GWAS which is typically much smaller, hence "missing heritability"). The non-genetic confounder is a composite of all other common causes of incidence and prognosis and as such we do not assume any single factor explaining 40% of variation. This was noted in the caption to figure 1, and we have now additionally clarified this at the top of page 5:

For a single SNP, we assume that incidence X is linear in the coded genotype G, the combined common causes U of incidence and prognosis, and causes E_X unique to X:

and page 17:

Recall that we assume incidence X is linear in the coded genotype G, the combined common causes U of incidence and prognosis, and causes E_X unique to X (equation 1):

While the authors admit that this is an unlikely scenario, they used it to be sure that the simulation "satisfies the assumptions of our procedure while creating a high degree of index event bias." Even with 65% of the variation in prognosis unexplained for each SNP (or explained by unmodeled confounders), the model is still unrealistic.

We simulate up to 65% of variation in prognosis explained by the combined causes in common with incidence. The effects of individual SNPs are much smaller, specifically 0.5/10,000 = 0.005% of variation in prognosis. This is now stated on page 20 paragraph 3:

For heritability of 50% distributed among 10,000 SNPs with effects on prognosis, each SNP explains, on average, 0.005% of variation. As half of SNPs affecting prognosis ~~which~~ also have effects on incidence, and the total non-genetic confounder variance is 40%, …

We have also clarified the descriptions of the simulation on page 7 paragraph 3:

Incidence and prognosis were simulated as quantitative traits under additive models with 50% heritability (Methods) ~~under equations (1) and (2) respectively.~~, with a non-genetic confounder (representing the combined effects of all such factors) simulated to explain 40% of variation in both incidence and prognosis.

and page 20 paragraph 4:

Incidence and prognosis traits were simulated from equations 1 and 2, with β_{GX} and β_{GX} now as the row vectors of effects for all SNPs, G as the column vector of genotypes and U consisting only of the non-genetic confounders.

Unfortunately, given this unrealistic scenario, the reader is unable to judge how important this issue is in the context of real world data. It would be useful to see some simulations for less extreme scenarios.

We accept that the degree of non-genetic confounding has a bearing on our performance, as well as the genetic confounding which we had already considered. We have therefore repeated the simulations with no net non-genetic confounding, in which case bias arises only through the genetic correlation. We now find that our method does worse when the genetic confounding is stronger than the non-genetic (page 8 paragraph 3):

We then repeated the simulation with no non-genetic confounding, so that index event bias only arises through genetic correlation violating our independence assumption. Table 3 shows that type-1 error for our approach is similar to that when non-genetic confounding is present, but for the unadjusted analysis the errors are reduced and generally closer to the nominal level than for our approach. Table 4 again shows a slight decrease in power under our approach, with considerable increases and decreases possible for individual SNPs. Supplementary tables 3 and 4 show similar patterns for absolute bias and mean square error.

However we argue that the situations in which our approach does better are the most plausible (page 8 paragraph 1, citing Paternoster et al [4], and page 8 paragraph 4):

…maintains the correct type-1 error rate when there is no genetic correlation between incidence and prognosis, and otherwise has a lower type-1 error rate than the unadjusted analysis except under strong negative genetic correlation or no non-genetic correlation. Of course, the relative

strength of genetic and non-genetic confounding is unknown in practice, but we might expect genetic and non-genetic confounding to act in the same direction, and the genetic confounding not to dominate the non-genetic. These are the scenarios in which our approach does best, and furthermore the type-1 errors are more consistent under different scenarios under our approach than the unadjusted analysis.

2. The models considered assume linearity between the outcomes and their predictors. However, the outcomes are inherently dichotomous (or survival outcomes). While the authors legitimately point out that the logistic model is nearly linear in portions of the predictor space, it would be more appropriate to address the underlying logistic model.

Some authors have quantified the index event bias under the logistic model [5,6] but their results do not lend themselves to our approach. Note that we use linear models to motivate our approach but then show that it is effective when the actual models are non-linear (page 15 paragraph 1):

This linear relationship is estimated from data, and while our theory provides an interpretation for it under some assumptions, our approach requires only that such a relationship exists. The data in our examples used log odds ratios and log hazard ratios, and our simulations suggested the linear approximation was acceptable in these cases.

3. It seems that a significance level of 5% is used in most evaluations. This alpha level seems unusually high for GWAS studies.

To address this we had evaluated the family-wise error over SNPs with effects on incidence but not on prognosis, of which there were 5000. This therefore evaluates the probability of at least one such SNP having $P < 10^{-5}$. Accurate evaluation of type-1 error for individual SNPs at such low error rates is computationally prohibitive.

Page 21 paragraph 1:

the proportion of simulations in which at least one SNP had $P < 0.05$ after Bonferroni adjustment for the number of such SNPs, that is $P < 10^{-5}$

Table 1 caption:

Family-wise error, probability of at least one SNP with effect on incidence but not on prognosis having $P < \frac{0.05}{5000} = 10^{-5}$.

4. Tables: the authors need to state what parameter/test the type I error and power are for. Is this the type I error and power for a single SNP test?

We have expanded the captions to table 1:

Table 1. Type-1 error (%) at $P < 0.05$ over 1000 simulations of 100,000 independent SNPs, adjusting for incidence as a quantitative trait. 5000 SNPs have effects on incidence only, 5000 on prognosis only and 5000 on both incidence and prognosis. Heritability of both incidence and prognosis is 50% with the genetic correlation shown over all SNPs. Common non-genetic factors explain 40% of variation in both incidence and prognosis. All SNPs, mean type-1 error over all SNPs. All SNPs affecting incidence, mean type-1 error over SNPs with effects on incidence but not on prognosis. SNP with highest error, individual SNP with effect on incidence but not on prognosis, having the

highest type-1 error under the respective analysis. Family-wise error, probability of at least one SNP with effect on incidence but not on prognosis having $P < \frac{0.05}{5000} = 10^{-5}$.

and table 2:

Table 2. Power (%) at $P < 0.05$ over 100,000 simulated independent SNPs, adjusting for incidence as a quantitative trait. Parameters and adjustments as in table 1. All SNPs, mean power over all SNPs. All SNPs affecting incidence, mean power over SNPs with effects on both incidence and prognosis. SNP with greatest increase (decrease) in power, individual SNP with effect on both incidence and prognosis having the greatest increase (decrease) in power in the adjusted analysis compared to the unadjusted.

5. The authors mention the important assumption of the independence between direct genetic effects on prognosis and those on incidence. Is there any way around this assumption? It seems likely that some genetic variants are associated with both incidence and prognosis. Does your approach mean that only variants that are different from (and indeed independent of, including those in LD) those identified for incidence are eligible for association with prognosis? Would conditioning on these variants associated with incidence in the prognosis analysis deal with this issue, allowing for newly discovered variants to those independent of the known ones for incidence? Further it would provide for evaluation of the known ones in regard to incidence.

We do allow for the same variants to have non-null effects on incidence and prognosis, but their effect sizes must be independent. This is a limitation of our method, but we believe our work opens up a new line of research on this problem, and we discuss some possible alternative approaches (page 13 paragraph 1):

Note though that this assumption applies only to the SNPs used in the regression of prognosis effects on incidence effects, after which the estimated adjustment may be applied to all SNPs. Independence could be assured, for example, by using only SNPs with no direct effect on prognosis. Identification of such SNPs, prior to bias adjustment, is not trivial but would be a worthwhile direction for further development.

and page 14 paragraph 4:

We may draw on the analogy with MR-Egger to contemplate other approaches based on the ratio of prognosis effects to incidence effects. Such approaches would entail other assumptions that require careful consideration. For example, a counterpart of the median ratio estimator would assume that at least half of the SNPs considered have no direct effect on prognosis.

Reviewer #2 (Remarks to the Author):

This is an interesting manuscript that discusses bias in genetic association test of a secondary phenotypes posterior to a primary index event (e.g. survival, severity). The authors propose solving the estimation of direct genetic effect on the secondary phenotype through an adjustment procedure. The problem is fairly complex but relatively well stated, and it seems that the proposed approach at least helps reducing the bias. However, the importance of the problem in real data and the performances of the method remain unclear (see major comments below). Moreover, the real data applications provide suggestive support at most, but highlight a limited contribution of the proposed approach in solving the problem. My major comments are:

1) It feels like the authors choose a modelling that fit their theoretical solution. It makes the interpretation of performances difficult. In particular, equation (2) assumes Y is a function of X. However, the final scenario of interest is slightly different -- Y is a phenotype that is measurable only among cases, i.e. there's no data for severity or survival among controls. It might be reasonable to build and demonstrate the relevance of the proposed solution using equation (2), but if the ultimate goal of the authors is to propose a solution applicable in real data, some changes are needed:

1a) the results from table 1 and 2 should be moved to the supplement. They are of limited interest as they relate to the approximation of equation (2).

We have retained tables 1 and 2 (and new tables 3 and 4) in the main text because 1) it is important to show that a method works under its own assumptions; 2) these tables show most clearly the pattern of results also apparent in the subsequent simulations; 3) this situation is the one described by Aschard et al [7], for which they were unable to identify a solution. Although we have presented our method mainly for prognosis in cases of disease, it has a wider range of application to which we should also draw attention (page 3 paragraph 3):

…although GWAS are often performed on prevalent cases, and our arguments also apply to selection or adjustment for a quantitative trait

Results from table 3) and 4) are not fully satisfying either as they also use equation (2). Instead the primary table(s) should present results from simulations where X is binary and generated using an LTM/logistic model, and Y is generated in cases only using a model that depends on G and U but not on X.

In fact this was what we had done. The simulation generated X from a liability threshold model (page 21 paragraph 5):

We simulated a binary selection event by treating the incidence trait as a liability with a threshold for disease such that 20% of individuals were affected.

and Y was generated from G and U but not X. We have now clarified this (page 7 paragraph 3):

No direct effect of incidence on prognosis was simulated ($\beta_{XY} = 0$).

1b) on top of effects of genetic correlation, it would be useful to have results showing the impact of disease prevalence, the distribution of the secondary outcome and minor allele frequency on the bias.

It is known that as the disease becomes rare, the bias vanishes so we deliberately chose prevalences (20% for incidence and 50% for prognosis) at the high end for complex disease.

Regarding the secondary outcome, although we had followed Paternoster et al [4] in simulating a normal trait (which could reflect a log-normal survival outcome), we do agree that non-normal traits would be more desirable. We have therefore replaced these simulations with ones where both incidence and prognosis traits are binary, the prognosis trait defined by thresholding a continuous latent variable at its median. We also performed simulations of a survival trait (see point 4 below). As the conclusions are similar to those from the normal trait, we have only retained the new simulations (page 8 paragraph 4):

Tables 5 to 8 ~~3 and 4~~ and supplementary tables 5 to 8 ~~3 and 4~~ show similar patterns when the incidence and prognosis traits are~~is a~~ binary ~~disease~~ and prognosis is analysed in cases only (Methods). They confirm that our approach is applicable under logistic regression ~~when incidence is analysed by logistic regression~~

Page 21 paragraph 5:

We then simulated a binary prognosis by thresholding the prognosis trait at its median, so that half the individuals had a good prognosis and half a poor prognosis. We obtained ~~and~~ unadjusted estimated effects on prognosis β'_{GY} from ~~linear~~ logistic regression of prognosis on genotype among cases only.

The effect of minor allele frequency has previously been described [5] and shows the intuitive property that for a fixed degree of bias, more common alleles yield higher type-1 errors (page 20 paragraph 5).

the index event bias is proportional to the effect on incidence and the rejection rate for a non-zero bias is greater for allele frequencies nearer 0.5.

We simulated a range of MAF across a GWAS but in practical terms the issue is whether our method works better for some SNPs than for others. We addressed this directly by identifying the individual SNPs with the greatest difference between unadjusted and adjusted analyses. These differences are driven not only by the MAF of the specific SNPs but also by their effect sizes. We think this is sufficient to show, qualitatively, the variation in performance across individual SNPs in addition to the average properties we present.

2) As showed in table 1 and 3, and as stated by the authors, the proposed method is imperfect, but likely reduces the bias as compared to a naïve approach that does not correct for anything. However, again, I feel the analysis is incomplete. In particular, as proposed by others, I ran a few simple simulations under the scenario described in comments 1a) and compared results of Y~G in cases only against Y~G+X in the complete sample (i.e. adjusting for the index event). Here I assumed Y is severity and positive only, setting Y to 0 for all controls. Overall, the latter solution perform clearly better than the case only analysis (i.e. most of the bias is removed). This solution is also imperfect as it can also induce a collider bias, but the question is, how much imperfect is it as compared to the proposed solution. Overall, my point is that the manuscript should include more method comparison, including the above strategy and any other potentially relevant approach.

We believe this would lengthen the paper unnecessarily. The $Y \sim G + X$ analysis is the one we used in the first simulation of continuous traits (page 20 paragraph 3):

Estimates of SNP effects on incidence $\hat{\beta}_{GX}$ were obtained from linear regression of incidence on genotype, and unadjusted estimates of SNP effects on prognosis $\hat{\beta}'_{GY}$ from linear regression of prognosis on genotype and incidence.

and now additionally at page 7 paragraph 3:

Incidence and prognosis were both analysed as quantitative traits with linear regression, with the prognosis model adjusting for incidence as a covariate.

This model can be used in conjunction with our approach to reduce bias for binary traits as you describe. It need not be an alternative to our approach, but one that can be used in conjunction with it, along with other strategies such as adjusting for the propensity score based on known confounders. A full comparison of different approaches is beyond the present scope, and highly dependent on context. For example, the Crohn's disease analysis used summary odds ratios calculated in cases only, so the $Y \sim G + X$ analysis simply cannot be applied in this case.

3) The main assumption of the method is the absence of correlation between SNP effect on incidence and on prognosis. At the same time –based on the authors work– bias exists only if there is an overlap of risk factors between incidence and prognosis (the U variables, explaining up to 40% of the outcome's variances in some simulations). If I understood correctly, we end up in the weird situation where the method is of interest when there is correlation in risk factors (and therefore some bias), but only when this correlation is not due to genetic factors. If so, I expect the actual real case scenarios where the method is valid and useful to be quite narrow.

More precisely, the assumption is that the correlation is not due to the total effect of the genetic factors used in the regression. We have clarified this on page 7, paragraph 2:

…no correlation between a SNP's effect on incidence β_{GX} and its direct effect on prognosis β_{GY}, for those SNPs entering the regression of step 2.

Although this is indeed a limitation we believe our work opens up a new line of research on this problem. In addition to the existing discussion on the parallels with MR (page 14 paragraph 4) we have added a new paragraph (page 13 paragraph 1):

Note though that this assumption applies only to the SNPs used in the regression of prognosis effects on incidence effects, after which the estimated adjustment may be applied to all SNPs. Independence could be assured, for example, by using only SNPs with no direct effect on prognosis. Identification of such SNPs, prior to bias adjustment, is not trivial but would be a worthwhile direction for further development.

4) The IPF result is not very convincing and I do not see it as a proof of concept – at most it suggests that the approach may be of interest in situations where the effect of a SNP on index event is strong. The reported variability of the parameters depending on the approach used and the imputation quality threshold, and the huge confidence interval are particularly concerning. Statement such as "[we] have shown an example in IPF where a strong effect on susceptibility leads to a substantial index event" do not reflect the actual reported result. The only fair conclusion from this analysis –and a guideline for future study- is, in my opinion, that the method is not applicable for small sample size.

We went to some length to challenge this result, including bootstrapping to test the direction of effect (page 9 paragraph 3), conditioning the effects in both GWAS on rs35705950 genotype (page

9 paragraph 4), and developing a more accurate version of the SIMEX algorithm (supplementary text 1). We were also circumspect about the interpretation, noting the imprecision and need for replication (which we are currently working towards):

Abstract:

we ~~resolved~~ reversed a paradoxical association of the strong susceptibility gene MUC5B with increased survival, ~~identifying~~ suggesting instead a significant association

Page 10 paragraph 1:

Together o~~O~~ur results suggest that the paradoxical association of MUC5B with increased survival could~~is~~ indeed be due to index event bias, and that the risk allele of MUC5B is in fact associated with decreased survival.

Page 12 paragraph 1:

an example in IPF where a strong effect on susceptibility appears to ~~leads to~~create a substantial index event bias that reverses the direction of the survival effect.

We have now, in addition, performed simulations of a survival prognosis trait, generated under the exponential model and analysed with Cox regression with the same sample size as our study (page 10 paragraph 1):

We repeated our earlier simulations using the reduced sample size of 612 cases and 3366 controls, generating survival times from the exponential model using the simulated prognosis trait as the log hazard, and testing association using Cox regression (supplementary tables 9 and 10). Adjusted and unadjusted analyses had similar overall properties, suggesting that our approach could be applicable in this setting.

In summary, while we accept the provisional nature of this result, we have been unable to identify any reason why it should be invalid; indeed the direction of effect is more intuitive than the unadjusted result.

Other comments:

5) In the modelling section, the authors state that the approximation of a logistic/LTM model is valid as long as the SNP effects are small ("the small effect typical to polygenic traits.", page 18). However, in their first simulation example, they consider strong effect ("[…] ensures that the individual SNP effects are strong given the heritability.", page 7). Are these effects strong enough to impact the validity of the approximation? A few words on this would is welcome.

This was the point of the simulations, which show that approximation does seem valid in practice. We have clarified this in the discussion (page 15 paragraph 1):

This linear relationship is estimated from data, and while our theory provides an interpretation for it under some assumptions, our approach requires only that such a relationship exists. The data in our examples used log odds ratios and log hazard ratios, and our simulations suggested the linear approximation was acceptable in these cases.

6) Page 6 – if I followed correctly, I think it should say "its effect on prognosis CONDITIONNAL ON X, beta_hat_prime[GY]…"

Thank you, we have inserted this text on page 6:

1. For each SNP, obtain an estimate of its effect on incidence $\hat{\beta}_{GX}$ with standard error σ_{GX}, and an estimate of its effect on prognosis $\hat{\beta}'_{GY}$ conditional on X with standard error σ_{GY}.

7) Page12 – Looks like a typo in the sentence "Some authors have argued that independently pleiotropic effects are likely to be the norm in complex disease". Should "independent" be removed?

No, we do mean that the pleiotropic effects are independent.

Reviewer #3 (Remarks to the Author):

Index event bias—which can occur when studying a trait that occurs after a primary event, notably when studying prognosis after disease diagnosis—is an important concern that often goes unacknowledged or is downplayed ("our study could be affected by index event bias, but the effect is probably small, and anyway there is nothing we can do about it"). This paper does a nice job explaining and highlighting this bias; placing it in the context of recent work on related issues (eg collider bias); and providing a clever procedure to actually adjust for index event bias. Through simulations and real data applications the authors show that their method can eliminate or reduce index event bias, and they give a feel for situations where this bias may be more or less of a concern.

One potential limitation is that the proposed methods are based on linear models for continuous phenotypes. This certainly makes the maths easier, and the authors show that the procedure performs reasonably well when applied to binary traits (notably disease incidence and prognosis). Nor is this the first time statistical geneticists apply least squared regression to non-continuous outcomes, decades of biostatistical tradition be damned. So this is fine—other subsequent papers can sort out whether other link functions help here.

Note that we only used least squares arguments to motivate the method, but in practice we do analyse binary data with logistic regression, and survival data with Cox regression, and show in simulations that our methods work acceptably in those situations. We clarified this in the discussion (page 15 paragraph 1)

The data in our examples used log odds ratios and log hazard ratios, and our simulations suggested the linear approximation was acceptable in these cases.

Another issue, which the authors do not discuss but should, is censoring. Survival after diagnosis is usually a censored trait, which opens up additional pathways to index event bias. I suspect that there are plenty of opportunities for both incidence and censoring to be affected by a common confounder—eg when studying lung cancer survival. SNPs associated with smoking will be associated both with lung cancer incidence and censoring (eg death due to cardiac disease). It may be that the methods and simulations developed for a direct incidence/prognosis confounder work fine when incidence and censoring are confounded, but some explicit discussion of this would be nice.

We had briefly mentioned survival effects (first paragraph of Discussion) but have now replaced this with a paragraph to clarify that they are not corrected by our current approach (page 15 paragraph 3):

Other forms of selection bias may be present that are not addressed by our approach. For example, participation in either incidence or prognosis GWAS is often conditional on survival until time of recruitment, but there may be unmeasured common determinants of survival and incidence/prognosis that create further biases. Genetic effects on incidence, estimated among survivors participating in GWAS, may not fully account for index event bias through our approach. We have previously shown survival bias to be potentially of similar importance to index event bias, and this should be borne in mind when performing studies of prognosis, particularly when the index event may be acute as in coronary heart disease.

A few minor points follow, that the authors may wish to address.

—investigators often construct a genetic risk score for incidence and then ask whether that is associated with prognosis. See eg PMID:24411283, Qi Guo. What are the implications of index event bias for these kind of analyses?

Anything associated with incidence is subject to index event bias, so this includes the genetic risk score for incidence. It can be corrected using our approach, if necessary treating the risk score like a major gene. We have added a sentence (top page 7):

A similar approach can be taken to polygenic scores aggregating the small effects of several individual SNPs.

—if one is interested in predicting prognosis, should one adjust for index event bias? As predictors, the "biased" estimates (in the sense of not capturing causal effects) may be just fine. This might merit a comment.

Yes indeed, we have added a paragraph (page 15 paragraph 5):

We have focussed on reducing bias in estimating the direct effects of SNPs on prognosis, to gain insight into mechanisms of prognosis. A different goal may be to build prediction models of prognosis. In that case it is preferable to work with the unadjusted effects since they do represent the total associations with prognosis conditional on incidence.

References

1. Stahl EA, Wegmann D, Trynka G, et al. Bayesian inference analyses of the polygenic architecture of rheumatoid arthritis. Nat Genet 2012;44:483-9.
2. Palla L, Dudbridge F. A Fast Method that Uses Polygenic Scores to Estimate the Variance Explained by Genome-wide Marker Panels and the Proportion of Variants Affecting a Trait. Am J Hum Genet 2015;97:250-9.
3. Vilhjalmsson BJ, Yang J, Finucane HK, et al. Modeling Linkage Disequilibrium Increases Accuracy of Polygenic Risk Scores. American Journal of Human Genetics 2015;97:576-92.
4. Paternoster L, Tilling K, Davey Smith G. Genetic epidemiology and Mendelian randomization for informing disease therapeutics: Conceptual and methodological challenges. PLoS Genet 2017;13:e1006944.
5. Yaghootkar H, Bancks MP, Jones SE, et al. Quantifying the extent to which index event biases influence large genetic association studies. Hum Mol Genet 2017;26:1018-30.
6. Sperrin M, Candlish J, Badrick E, Renehan A, Buchan I. Collider Bias Is Only a Partial Explanation for the Obesity Paradox. Epidemiology 2016;27:525-30.
7. Aschard H, Vilhjalmsson BJ, Joshi AD, Price AL, Kraft P. Adjusting for heritable covariates can bias effect estimates in genome-wide association studies. Am J Hum Genet 2015;96:329-39.

Reviewer #1 (Remarks to the Author):

I am satisfied with the responses and the changes to the manuscript.

Reviewer #2 (Remarks to the Author):

The authors replied to most of my comments. While I do not always agree, I think the responses are fair and address my concerns. However, they did not reply to comment 2), which I think is critical to determine the practical utility of the proposed method.

In this comment I questioned the relevance of the proposed method in comparison with the existent. In particular I asked that the results from (now) tables 5 and 7 to be generated for the model Y ~ G+X, where the severity Y is set at zero for controls, instead of looking at Y ~ G in case only data. The authors replied that ”We believe this would lengthen the paper unnecessarily. The Y~G + X analysis is the one we used in the first simulation of continuous traits (page 20 paragraph 3):”

I disagree and I believe that such analysis will be a key guideline for using (or not) their approach in real data analysis. Indeed, while they claim that some studies have case only data (or summary results from case-only analysis), many others have also controls. For those studies, having a comparison between the authors approach and adjusting for case-control status will be of major importance. From my perspective, adding such a simulation should be easy and fast, and will definitely not “unnecessarily lengthen the paper”.

In short, I can clearly see that for continuous variable, the proposed method displays on average lower bias than the Y~G + X approach (table 1) as long as there are common non-genetic factors of incidence and prognosis (table 3). For binary outcome and using case only, the pattern is similar, but the differences are much more attenuated (Table 5). Based on the toy simulation I ran at the previous review stage, it seems possible that the Y~G + X model perform better than the approach proposed by the author. If so, it would reduce the scope of interest of the method to situation where cohort have cases only, and make it of limited interest otherwise. If Y~G + X model is not doing better, that would also be of great interest for investigators, offering thus a better alternative that naïve adjustment for incidence.

Other minor comment.

Point 7. (Page12 – Looks like a typo in the sentence “Some authors have argued that independently pleiotropic effects are likely […] are independent.’)

I think, but not 100% sure to understand. Does it means that e.g. taking multiple SNPs, that are associated with two outcomes A and B (i.e. therefore pleiotropic) with effects defines by the vectors bA and bB, we would not observed correlation of between bA and bB? Whatever the exact meaning, it is worth expending a bit this sentence to make it clearer.

Reviewer #3 (Remarks to the Author):

The authors have mostly addressed my comments. But they may not have fully understood my comment on censoring after diagnosis (i.e. after incidence). The new paragraph starting "Other forms of selection bias..." addresses an important point (which they give me too much credit for thinking I was smart enough to raise)--namely, ascertainment bias due to survival bias. But this is not what I was getting at. I'm worried about association between G and censoring after incidence. So in the lung cancer case, b_GY might be 0, where Y is time to death due to lung cancer, but what happens if b_GC is !=0, where C is censoring (i.e. death due to heart attack). This will violate the assumption of independent censoring.

I don't think this is a major concern, it may just need a short note. The independence of censoring from the exposures of interest is (I believe) a standard assumption in Cox regression (e.g.)--it probably suffices just to remind the reader what can happen if that assumption is violated (and that the proposed procedure does not [?] adjust for that violation).

Adjustment for index event bias in genome-wide association studies of subsequent events

Response to second reviews

Reviewer #2 (Remarks to the Author):

The authors replied to most of my comments. While I do not always agree, I think the responses are fair and address my concerns. However, they did not reply to comment 2), which I think is critical to determine the practical utility of the proposed method.

In this comment I questioned the relevance of the proposed method in comparison with the existent. In particular I asked that the results from (now) tables 5 and 7 to be generated for the model Y ~ G+X, where the severity Y is set at zero for controls, instead of looking at Y ~ G in case only data. The authors replied that "We believe this would lengthen the paper unnecessarily. The Y~G + X analysis is the one we used in the first simulation of continuous traits (page 20 paragraph 3):"

I disagree and I believe that such analysis will be a key guideline for using (or not) their approach in real data analysis. Indeed, while they claim that some studies have case only data (or summary results from case-only analysis), many others have also controls. For those studies, having a comparison between the authors approach and adjusting for case-control status will be of major importance. From my perspective, adding such a simulation should be easy and fast, and will definitely not "unnecessarily lengthen the paper".

In short, I can clearly see that for continuous variable, the proposed method displays on average lower bias than the Y~G + X approach (table 1) as long as there are common non-genetic factors of incidence and prognosis (table 3). For binary outcome and using case only, the pattern is similar, but the differences are much more attenuated (Table 5). Based on the toy simulation I ran at the previous review stage, it seems possible that the Y~G + X model perform better than the approach proposed by the author. If so, it would reduce the scope of interest of the method to situation where cohort have cases only, and make it of limited interest otherwise. If Y~G + X model is not doing better, that would also be of great interest for investigators, offering thus a better alternative that naïve adjustment for incidence.

In our revised paper, the simulation with binary incidence had a binary rather than a continuous prognosis (tables 5 to 8). If we now assign a fixed prognosis to all controls, we cannot fit the model Y~G+X with logistic regression since the log-odds of that prognosis given X=0 are infinite.

But reverting to our original situation with continuous prognosis, we were able, like the reviewer, to simulate a simple scenario with one SNP explaining 50% of variation, where Y~G+X had much less bias than Y[case]~G[case]. However, this behaviour is not universal; for example the simulations of Monsees et al (ref 18) showed the case-only analysis to have less bias than Y~G+X (their table 3 and figure 5). We have applied the Y~G+X analysis to the GWAS simulations of our paper, setting Y to 0 in controls. In these simulations, bias is averaged over thousands of SNPs with small effects, and we found essentially no difference in results between the Y~G+X analysis and the case-only analysis (supplementary tables 9 to 12). We could not find a situation, in the

GWAS context, in which Y~G+X should be preferred to both our approach and case-only analysis. Of course that is not to say that such a situation does not exist.

We have added the following text (page 8 para 4):

Tables 5 to 8 and supplementary tables 5 to 8 show similar patterns when the incidence and prognosis traits are binary and prognosis is analysed in cases only (Methods). Supplementary tables 9 to 12 also show similar patterns when the prognosis is quantitative and analysed either in cases only or in the full sample with adjustment for case/control status (Methods). These~~y~~ results confirm that our approach is applicable ~~under~~ when incidence is analysed by logistic regression

(Page 15 para 2):

When the incidence trait is binary, we have mainly considered a case-only analysis of prognosis. Other approaches are possible, such as setting the prognosis to a degenerate value for controls and then analysing cases and controls together, with adjustment for case/control status [18]. Although this approach still creates a collider bias, it may be less severe in some situations than the case-only analysis, and would then compare more favourably with our approach. However, in our simulations we found no systematic difference between the case/control and case-only analysis. Note that our approach could be applied in conjunction with the case/control analysis, and possibly with further adjustment for measured potential confounders of incidence and prognosis. This would have the desirable effect of reducing index event bias through several complementary approaches at once.

(Page 21 para 6):

For the binary selection event we also analysed the prognosis trait on its original quantitative scale using linear regression of prognosis on genotype among cases only, and compared results to the analysis of the combined case/control sample with statistical adjustment for case/control status, imputing a value of 0 for prognosis among controls. The latter approach may, in some situations, lead to reduced bias or increased power in comparison with case only analysis [18].

Other minor comment.

Point 7. (Page12 – Looks like a typo in the sentence "Some authors have argued that independently pleiotropic effects are likely […] are independent.')

I think, but not 100% sure to understand. Does it means that e.g. taking multiple SNPs, that are associated with two outcomes A and B (i.e. therefore pleiotropic) with effects defines by the vectors bA and bB, we would not observed correlation of between bA and bB? Whatever the exact meaning, it is worth expending a bit this sentence to make it clearer.

Yes this is the correct understanding. We have expanded the sentence as follows (bottom page 12):

Some authors have argued that independently pleiotropic effects are likely to be the norm in complex disease [25]: for most pairs of traits the genetic effects on the first are independent of the corresponding genetic effects on the other.

Reviewer #3 (Remarks to the Author):

The authors have mostly addressed my comments. But they may not have fully understood my comment on censoring after diagnosis (i.e. after incidence). The new paragraph starting "Other forms of selection bias..." addresses an important point (which they give me too much credit for thinking I was smart enough to raise)--namely, ascertainment bias due to survival bias. But this is not what I was getting at. I'm worried about association between G and censoring after incidence. So in the lung cancer case, b_GY might be 0, where Y is time to death due to lung cancer, but what happens if b_GC is !=0, where C is censoring (i.e. death due to heart attack). This will violate the assumption of independent censoring.

I don't think this is a major concern, it may just need a short note. The independence of censoring from the exposures of interest is (I believe) a standard assumption in Cox regression (e.g.)--it probably suffices just to remind the reader what can happen if that assumption is violated (and that the proposed procedure does not [?] adjust for that violation).

Informative censoring can certainly create bias that is not addressed by our approach. Indeed it need not relate to the index event at all, and there are numerous other factors that can bias associations with prognosis (measurement error, competing risks etc). With other collaborators we are currently working on elucidating all the relevant scenarios. We have noted the reviewer's point in the following additional text (page 16):

Censoring after diagnosis, for example from death by competing risks, may also create bias if there are common determinants of incidence, censoring and/or prognosis. Our approach is developed under a simple model of incidence and prognosis, but provides a starting point for extensions that model the disease course more precisely.

Reviewer #2 (Remarks to the Author):

I appreciate the additions to the manuscript, and I do not have any further comments.
